# Transforming growth factor β1 promotes invasion of human JEG-3 trophoblast cells via TGF-β/Smad3 signaling pathway

**DOI:** 10.18632/oncotarget.16826

**Published:** 2017-04-04

**Authors:** Zhongying Huang, Shangwei Li, Wei Fan, Qianhong Ma

**Affiliations:** ^1^ Department of Obsterics and Gynecology, West China 2nd Hospital, University of Sichuan, Key Laboratory of Birth Defects and Related Diseases of Women and Children, Ministry of Education, Chengdu 610041, People's Republic of China

**Keywords:** TGF-β signaling, Smads, matrix metalloproteinases, trophoblast

## Abstract

Transforming growth factor (TGF)-β1 is involved invasion of human trophoblasts. However, the underlying mechanisms remain unclear. In this study, we performed Transwell assay and found that TGF-β1 promoted the invasion of trophoblast cell line JEG-3. Treatment with TGF-β1 up-regulated the expression of receptor-regulated Smad transcription factors Smad2 and Smad3, and two invasive-associated genes, namely, matrix metallopeptidase (MMP)-9 and MMP-2, in JEG-3 cells. Over-expressing activin receptor-like kinase (ALK) 5, the TGF-β type I receptor (TβRI) enhanced the up-regulation of Smad2, Smad3, MMP-9, and MMP-2 induced by TGF-β1, whereas application of TβRI inhibitor SB431542 diminished the stimulatory effects of TGF-β1 on these genes. Furthermore, transfection of Smad3 and ALK-5 seperately or in combination into JEG-3 cells before TGF-β1 treatment significantly increased the expression of MMP-9 and MMP-2. By contrast, silencing Smad3 and Smad2 by siRNAs significantly decreased the expression of MMP-9 and MMP-2, with Smad3 silence having a more potent inhibitory effect. Inhibiting TβRI with SB431542 or knockdown of Smad3, but not Smad2, abolished the stimulatory effect of TGF-β1 on the invasion of JEG-3 cells. Taken together, the results indicate that TGF-β1 activates the Smads signaling pathway in JEG-3 trophoblast cells and Smad3 play a key role in TGF-β1-induced invasion of JEG-3 and up-regulation of MMP-9 and MMP-2 expression.

## INTRODUCTION

Establishment of a functional placenta is pivotal for normal fetal development and maintenance of pregnancy. Invasion of extravillous trophoblasts (EVTs) into the maternal decidua and inner myometrium is essential for successful human placental development and pregnancy progression [1–3]. Various severe pregnancy complications, such as preeclampsia or fetal growth restriction, are associated with abnormal EVT function, shallow invasion, and decreased blood flow to the placenta [4–7]. Mechanisms underlying trophoblast invasion must be elucidated to improve therapeutic interventions. The invasion of trophoblasts is regulated by a complex network of cell types, mediators, growth factors, cytokines, various matrix metalloproteinases and their inhibitors, and intracellular signaling pathways [8–11].

Transforming growth factor-β1 (TGF-β1) is a multifunctional cytokine that regulates various cellular functions, including cell proliferation, differentiation, apoptosis, migration, matrix synthesis, and immune responses [12–17]. TGF-β1 has also been suggested to be involved in the invasion of human trophoblasts [18–21]. Nevertheless, the molecular mechanisms through which TGF-β1 affects the invasion of trophoblasts have not been elucidated yet.

Previous studies indicated that TGF-β1 signals through Smad-dependent (canonical) and Smad-independent (non-canonical) pathways [22–26]. The Smad pathway is a primary mediator of TGF-β signaling [27]. The cellular activities of TGF-β are mediated by specific receptor complexes; these complexes are assembled upon ligand binding and consist of TGF-b type II receptor (TβRII) and TGF-β type I receptor (TβRI/ALK5). The activated ligand–receptor complex typically activates the Smad signaling pathway, also called the canonical Smad pathway. This pathway is initiated by activating ALK5 through C-terminal phosphorylation of receptor-regulated Smad transcription factors (R-Smads), such as Smad2 and/or Smad3; the activated ALK5 form complexes with the Co-mediator Smad (Co-Smad, namely Smad4) and accumulates in the nucleus, binds to DNA, and regulates the transcription of target genes [27–29].

The role of the TGF-β/Smad pathway in regulating trophoblast cell invasion remains unclear. Several recent studies on other cell types reported the involvement of the canonical Smad pathway in TGF-β-induced cell invasion [30–32]. Therefore, we hypothesize that a similar pathway might be involved in the action of TGF-β1 on the invasion of trophoblast cells. In the present study, we examined the effects of TGF-β1 on the invasion of trophoblast cells and investigated the underlying signaling mechanisms by using the EVT cell model JEG-3.

## RESULTS

### TGF-β1 promotes the invasion of trophoblast cell line JEG-3

**Figure 1:**
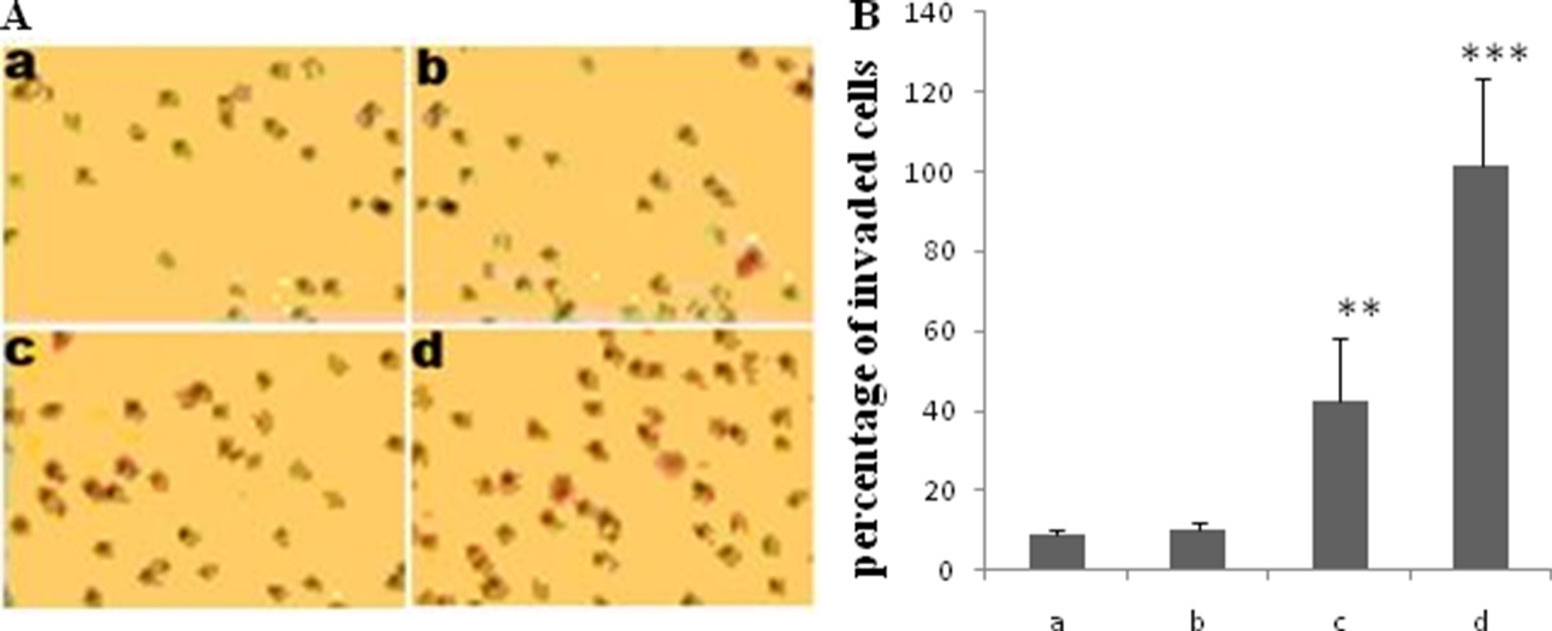
Effect of TGF-β1 on invasion of JEG-3 cells Cells were treated with TGF-β1 at different concentration: 0 ng/ml (a, the control), 5 ng/ml (b), 10 ng/ml (c) and 20 ng/ml (d). Transwell assay was performed to determine the effect of TGF-β1 on JEG-3 cell invasion. (**A)** The invaded cells were visualized 24 h following treatment with TGF-β1 by staining with a 0.1% crystal violet solution. (**B**) The invaded cells significantly increased with TGF-β1 treatment at the concentration of 10 and 20 ng/ml. Data were represented as mean ± SD. Three independent experiments were performed with each condition being tested in triplicate. **P < 0.01 vs the control; ***P < 0.001 vs the control.

In this study, Transwell assay was conducted to evaluate the effect of TGF-β1 on the invasion ability of JEG-3 cells. The cells were treated with different concentrations of TGF-β1 (0, 5, 10, and 20 ng/ml). The number of invaded cells was significantly increased following treatment with 10 or 20 ng/ml TGF-β1 (Figure 1A and 1B).

### TGF-β1 up-regulates the expression of MMP-9 and MMP-2 in JEG-3 cells

**Figure 2:**
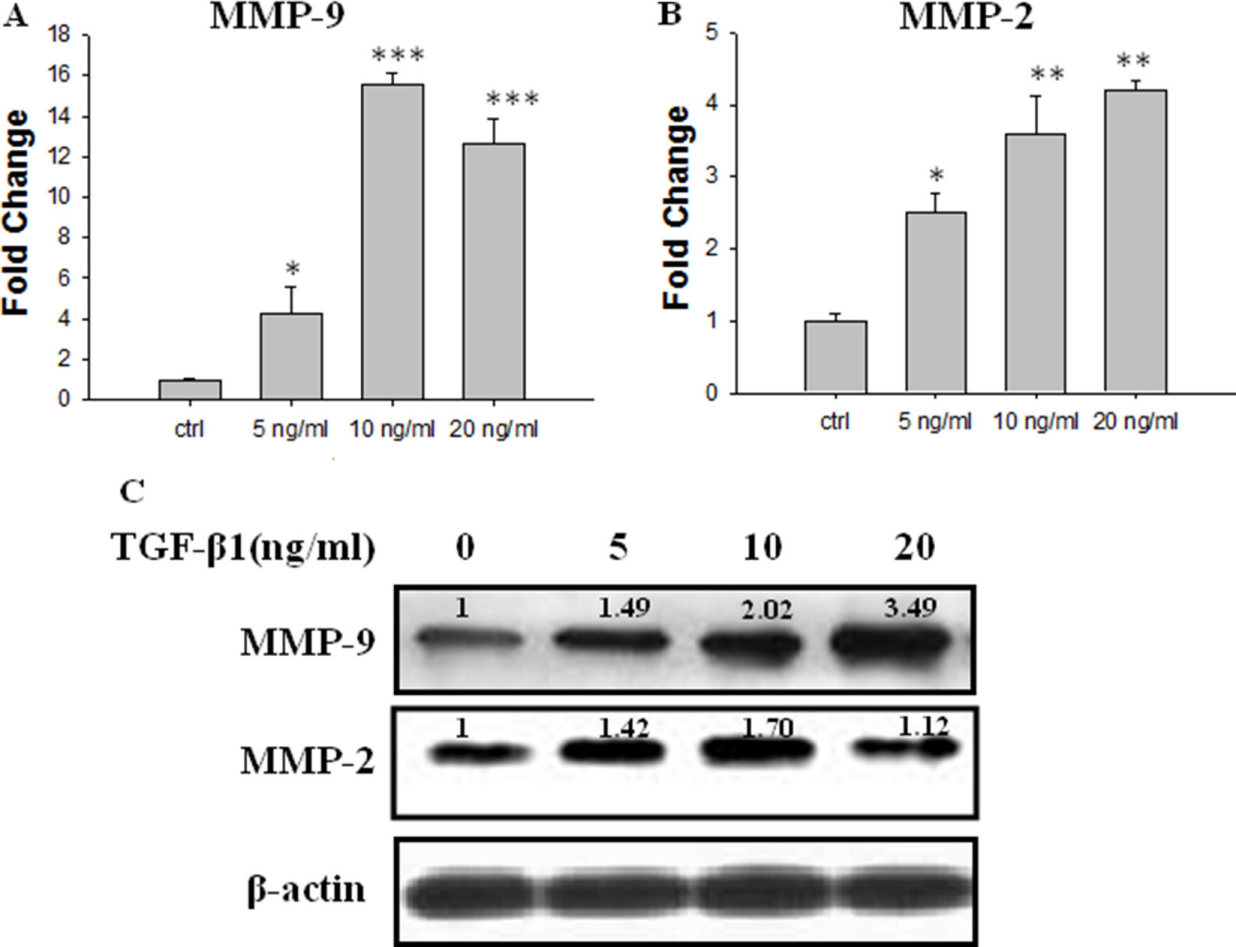
Effect of TGF-β1 on the expression of MMP-2 and MMP-9 in JEG-3 cells. Cells were treated with TGF-β1 at different concentration: 0 ng/ml (the control), 5 ng/ml, 10 ng/ml and 20 ng/ml. (**A**) Real-time PCR analysis of MMP-9 mRNA 48 h following treatment with TGF-β1. (**B**) Real-time PCR analysis of MMP-2 mRNA 48h following treatment with TGF-β1. (**C**) Western blot analysis of MMP-9 and MMP-2 mRNA 48 h following treatment with TGF-β1. Three independent experiments were performed with each condition being tested in triplicate.*P < 0.05 vs the control; **P < 0.01 vs the control; ***P < 0.001 vs the control.

The real-time PCR data showed that TGF-β1 treatment concentration-dependently increased the mRNA levels of MMP-9 and MMP-2 (Figure 2A and 2B). Treatment with TGF-β1 (10 ng/ml) for 48 h increased the MMP-9 mRNA expression level by approximately 16-fold compared with that in the control (Figure 2A). The Western Blot assay revealed that treatment with TGF-β1 at or above 5 ng/ml significantly increased the protein expression levels of MMP-9. Meanwhile, there is an increase tendency of MMP-2 protein, but without any statistic significance (Figure 2C).

### Effect of TGF-β1 on Smads expression in JEG-3 cells

**Figure 3:**
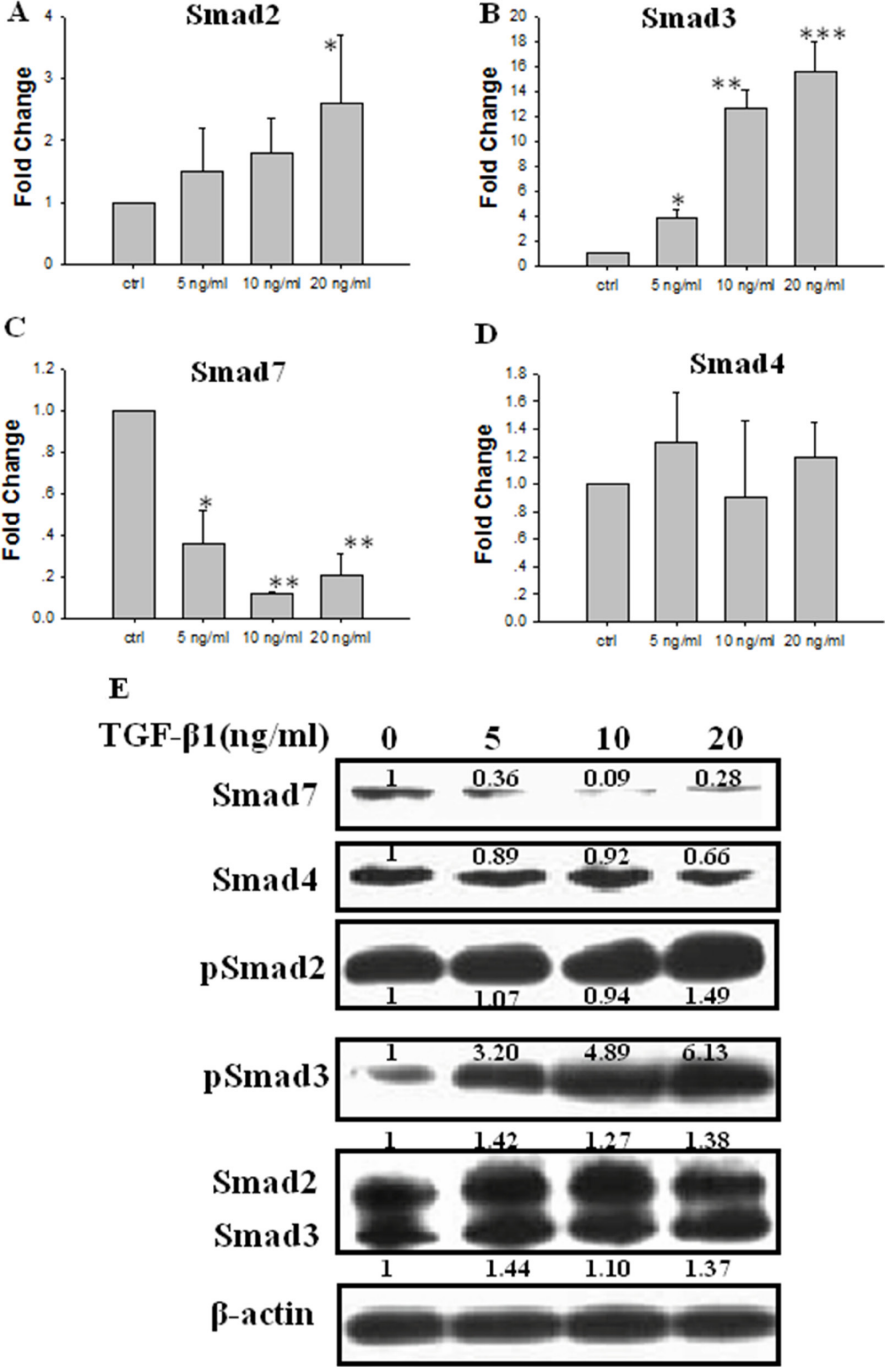
Effect of TGF-β1 on the expression of Smads in JEG-3 cells Cells were treated with TGF-β1 for 48 h at different concentration: 0 ng/ml (the control), 5 ng/ml, 10 ng/ml and 20 ng/ml. (**A**) Real-time PCR analysis of Smad2 mRNA following treatment with TGF-β1. (**B**) Real-time PCR analysis of Smad3 mRNA following treatment with TGF-β1. (**C**) Real-time PCR analysis of Smad7 mRNA following treatment with TGF-β1. (**D**) Real-time PCR analysis of Smad4 mRNA following treatment with TGF-β1. (**E**) Western blot analysis of Smad2, 3, 4, 7, pSmad2 and pSmad3 following treatment with TGF-β1. Three independent experiments were performed with each condition being tested in triplicate. *P < 0.05 vs the control; **P < 0.01 vs the control; ***P < 0.001 vs the control.

JEG-3 cells were treated with different concentrations of TGF-β1 for 48 h. Real-time PCR data showed that the mRNA levels of Smad2 in JEG-3 cells gradually increased with increasing TGF-β1 concentration and significantly increased upon treatment with 20 ng/ml TGF-β1 (Figure 3A). The mRNA expression of Smad3 significantly increased in a concentration-dependent manner after treatment with TGF-β1 (Figure 3B). The mRNA level of Smad7 significantly decreased following TGF-β1 treatment (Figure 3C). The Smad4 mRNA levels was not significantly different from that in the control (Figure 3D). The protein expression levels of Smad2 or Smad3 were not significant differed between cells treated with TGF-β1. The expression level of the phosphorylated Smad2 (pSmad2) slightly increased when the cells were treated with 20 ng/ml TGF-β1. Moreover, the expression of phosphorylated Smad3 (pSmad3) was significantly stimulated by treatment with TGF-β1 at or above 5 ng/ml in a concentration-dependent manner. By contrast, the protein levels of Smad7 decreased significantly following TGF-β1 treatment in concentration-dependent manner. No significant differences were observed in the protein expression of Smad4 following TGF-β1 treatment (Figure 3E).

### Involvement of TGF-β/Smads pathway in JEG-3 cell invasion

**Figure 4:**
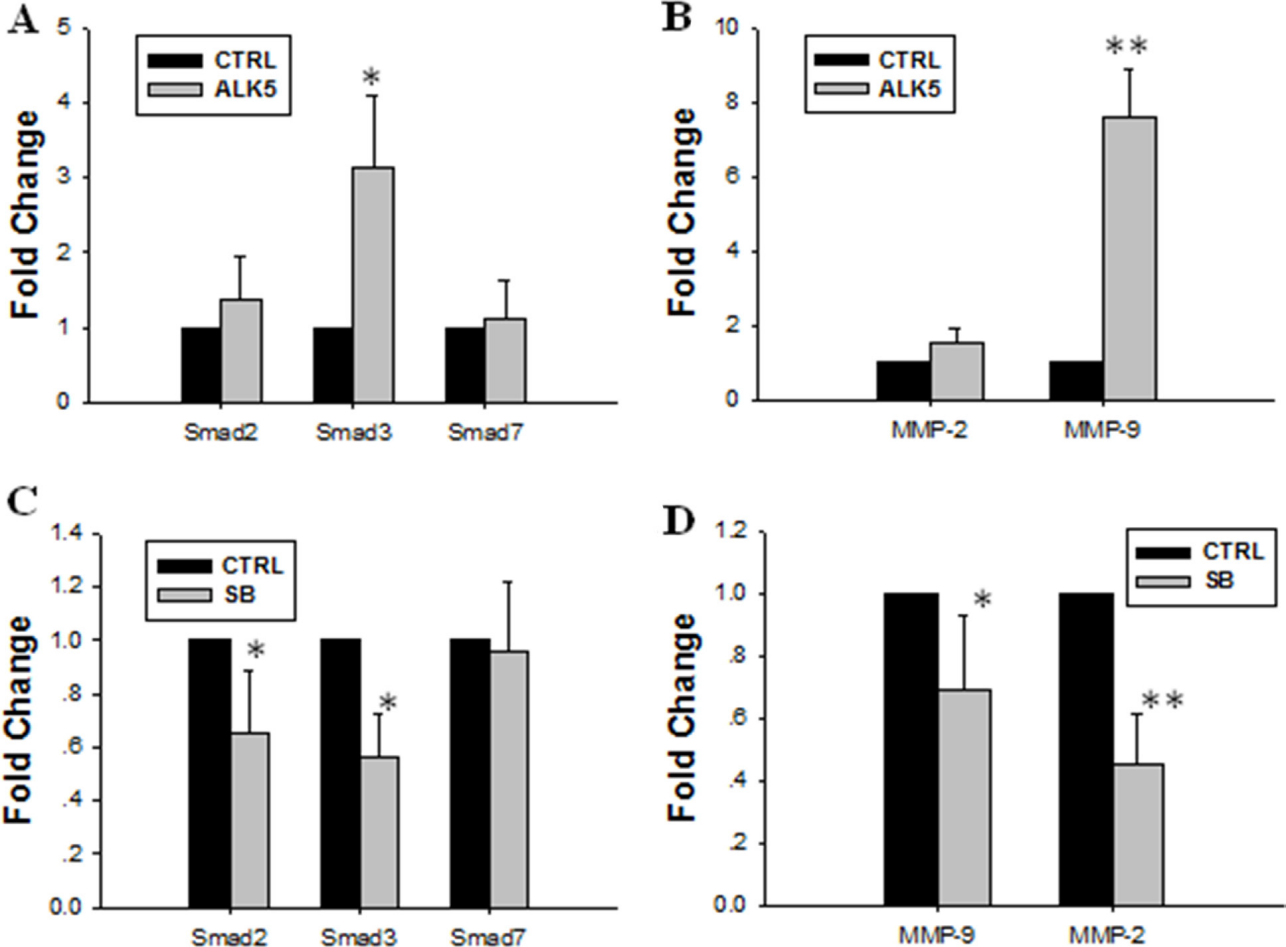
Effect of over-expression or inhibition of ALK5 on expression levels of Smad2, Smad3, MMP-9 and MMP-2 (**A)** ALK5 transfection caused significant increase of Smad3 mRNA expression level. (**B**) ALK5 transfection caused significant increase of MMP-9 mRNA expression. (**C)** Inhibition of ALK5 with SB431542 abolished the stimulatory effect of TGF-β1 on the mRNA expression levels of Smad2 and Smad3. (**D**) Inhibition of ALK5 with SB431542 diminished the stimulatory effect of TGF-β1 on expression levels of MMP-9 and MMP-2. Data were represented as mean ± SD. Three independent experiments were performed with each condition being tested in triplicate. *P < 0.05 vs the control; **P < 0.01 vs the control.

In this study, ALK5 overexpression by plasmid transfection increased the mRNA expression of Smad3. However, the expression of Smad2 or Smad7 mRNA in transfected cells was not significantly different from that in control cells (Figure 4A). Over-expressing ALK5 also increased the mRNA expression of MMP-9 (Figure 4B). Pretreatment with SB431542, a potent and selective inhibitor of ALK5, diminished the stimulatory effect of TGF-β1 on the expression of Smad2 and Smad3 in JEG-3 cells but did not influence that of Smad7 (Figure 4C). Treatment with SB431542 also abolished the stimulatory effect of TGF-β1 on the expression of MMP-9 and MMP-2 (Figure 4D).

**Figure 5:**
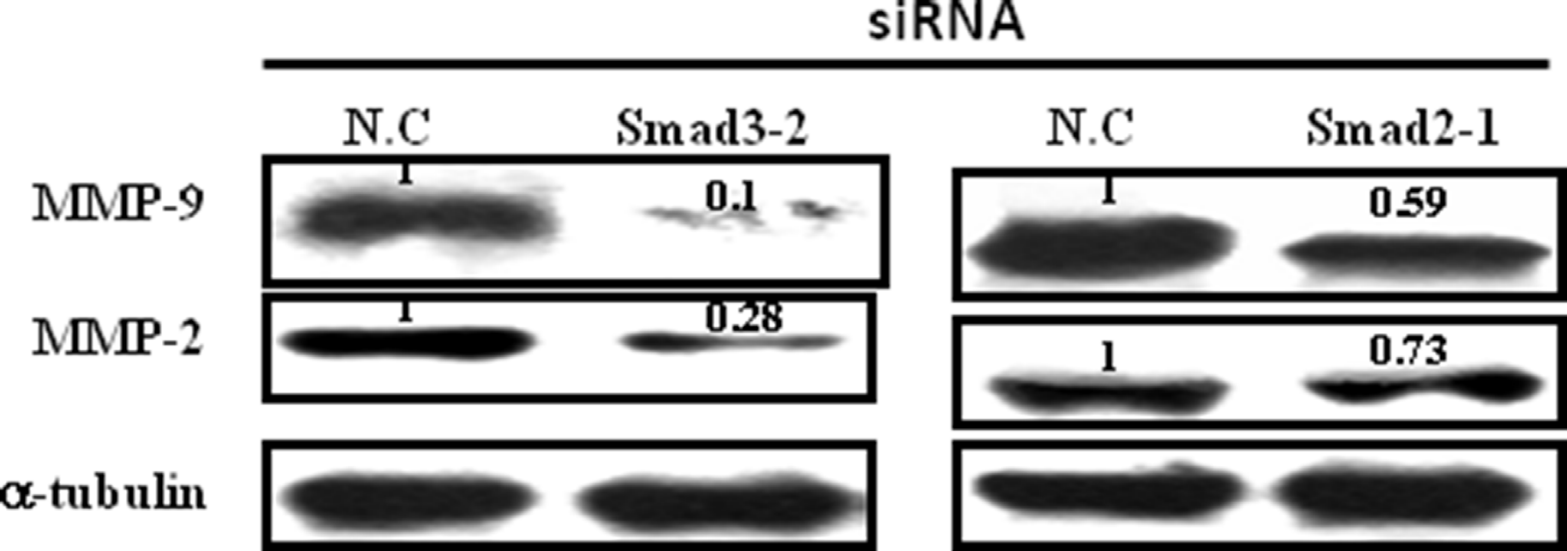
Effect of Smad2 and Smad3 silence on the expression of MMP-9 and MMP-2 JEG-3 cells were transfected with siRNAs targeting Smad2 or Smad3, respectively. Silence of both Smad2 and Smad3 decreased the expression levels of MMP-9 and MMP-2, with most potent inhibition efficacy following Smad3 siRNA transfection.

Smad2 and Smad3 were silenced using RNAi technology to examine the roles of endogenous Smad2 and Smad3 in the regulation of MMP-9 and MMP-2 expression in JEG-3 cells. Small-interfering RNAs (siRNAs) targeting Smad2 and Smad3 genes were designed and evaluated. JEG-3 cells were transfected with siRNAs targeting Smad2 (siRNA Smad 2-1, -2, -3) and Smad3 (siRNA Smad 3-1, -2, -3). Among them, siRNA Smad 2-1 and siRNA Smad3-2 exhibited the most potent interference efficacy on blocking Smad2 and Smad3 protein expression (Supplementary Figure 1). siRNA Smad 2-1 and siRNA Smad 3-2 were transfected into JEG-3 cells respectively. Data showed that silencing Smad2 or Smad3 by siRNAs transfection inhibited the expression of MMP-9 and MMP-2 in JEG-3 cells; transfection with Smad3-2 siRNA exhibited the most potent inhibition efficacy (Figure 5).

**Figure 6:**
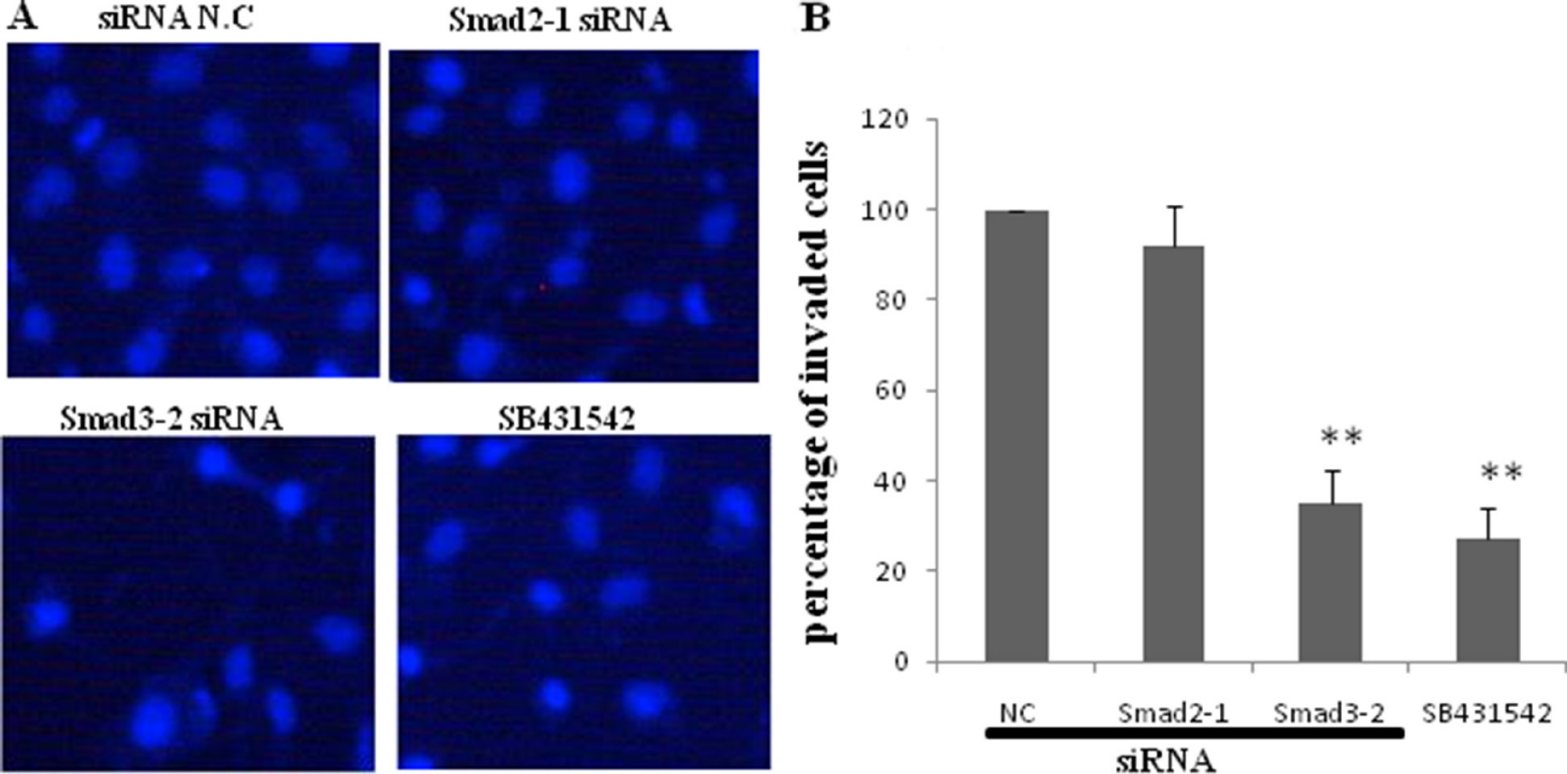
Effect of blockage of TGF-β1/Smads signal pathway on invasion of JEG-3 JEG-3 cells were transfected with siRNA negative control, siRNA Smad2-1, and siRNA Smad3-2 respectively or pretreated with SB431542, before subjected to TGF-β1. (**A**) Transwell assay was performed and invaded cells were visualized 24 h following treatment by staining with DAPI. (**B**) SB431542 significantly diminished the effect of TGF-β1 on JEG-3 invasion. Knockdown of Smad3 resulted in a remarkable decrease of cell invasion. The silence of Smad2 did not cause significant difference in the numbers of invaded cells compared with the control. Data were represented as mean ± SD. Three independent experiments were performed with each condition being tested in triplicate. **P < 0.01 vs the control.

JEG-3 cells were transfected with siRNA negative control, siRNA Smad 2-1, and siRNA Smad 3-2 or pretreated with SB431542 before treatment with TGF-β1 to understand the roles of endogenous Smad2 and Smad3 in JEG-3 cell invasion. The cells were subjected to Transwell assay. Results showed that TGF-β1-induced JEG-3 invasion was significantly reduced with SB431542 treatment. Knockdown of Smad3 by siRNA transfection remarkably reduced cell invasion, comparable with the effect of SB431542. Silencing Smad2 did not significantly change the number of invaded cells compared with the control (Figure 6A and 6B).

**Figure 7:**
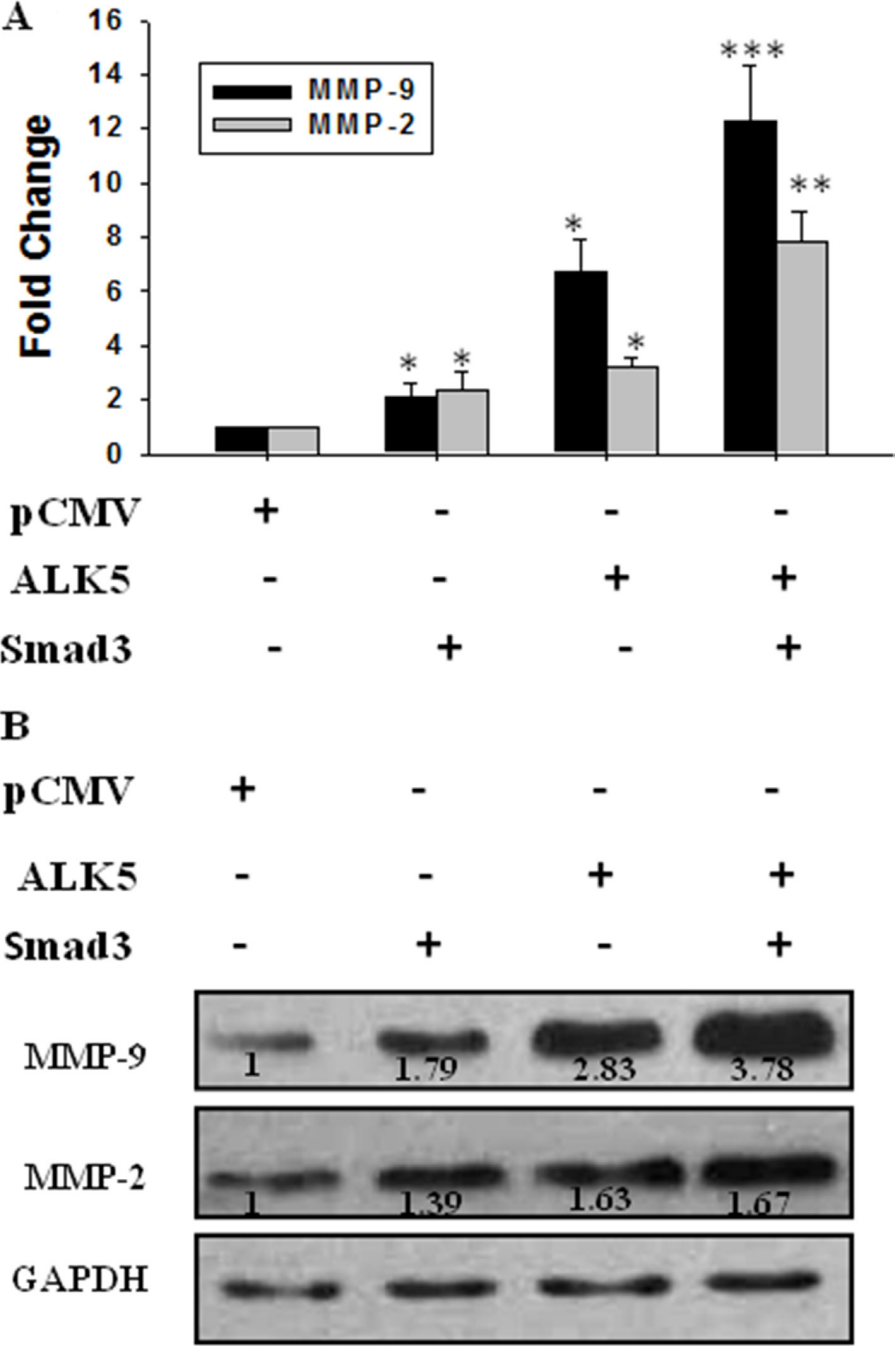
Effect of activation of TGF-β/Smad3 signal pathway on expression of MMP-9 and MMP-2 JEG-3 cells were transfected with pCMV (the control), ALK5, and Smad3 or co-transfected with ALK5 and Smad3 before treatment with TGF-β1. (**A**) Real-time PCR showed that over-expression of both Smad3 and ALK5 up-regulated MMP-9 and MMP-2. The co-transfection of ALK5 and Smad3 increased the MMP-9 and MMP-2 levels significantly. (**B**) The protein levels were detected using Western blot. Over-expression of both Smad3 and ALK5 increased the expression levels of MMP-9 and MMP-2, with most potent stimulation efficacy following co-transfection of ALK5 and Smad3. Data were represented as mean ± SD. Three independent experiments were performed with each condition being tested in triplicate. *P < 0.05 vs the control; **P < 0.01 vs the control; ***P < 0.001 vs the control.

JEG-3 cells were transfected with pCMV5, ALK5 and Smad3, or ALK-5 co-transfected with Smad3 before treatment with TGF-β1 to further demonstrate the effect of Smad3. Over-expressing Smad3 and ALK5 up-regulated the mRNA expression of MMP-9 and MMP-2. When the cells were co-transfected with ALK-5 and Smad3, the expression levels of MMP-9 and MMP-2 mRNA were up-regulated by about 12- and 8-fold, respectively (Figure 7A). Western blot analysis revealed a similar change tendency in the MMP-9 and MMP-2 protein expression levels (Figure 7B).

## DISCUSSION

TGF-β/Smad signaling pathway is involved in various cell processes under physiological and pathological conditions. The role of TGF-β1 in regulating the invasion of human trophoblasts has been studied for years [18–21, 33–34]. However, whether TGF-β1 signaling signals through Smad pathway to regulate trophoblast invasion and the underlying molecular mechanisms remains unclear. In this study, a well-established cellular model for EVTs, namely, JEG-3 cells, was used to demonstrate that TGF-β1 promotes the invasion of JEG-3 via the Smad3 signaling pathway.

In the present study, R-Smads, namely, Smad2 and Smad3, in JEG-3 cells were up-regulated following TGF-β1 treatment. Our findings are partly in agreement with a report that TGF-β1 increased Smad2 mRNA levels without affecting Smad3 mRNA expression [33]. The stimulatory effect of TGF-β1 on Smad2 and Smad3 expression was enhanced by over-expressing ALK5 but blocked by the TβRI inhibitor SB431542. These results are consistent with those reported in other cell types; that is, TGF-β1 signals via TβRI activated the downstream transduction effectors Smad2/3 (p-Smad2/3) [27, 28, 35]. The expression level of the inhibitory Smad7 decreased following TGF-β1 treatment. Smad7 competes with R-Smads for receptor binding, thereby inhibiting R-Smad phosphorylation [28, 29]. Hence, the decreased expression of Smad7 may alleviate the inhibitory effects on phosphorylation of Smad2/3, which partly contribute to the activation of the Smad2/3 signal pathway. In the current study, the Smad4 expression level unchanged. Further research may be needed to elucidate the precise mechanism of Smad4 action in this process.

TGF-β1 increased the invasive ability of JEG-3 cells and the expression levels of Smad2 and Smad3; hence, TGF-β1 promotes the invasion of JEG-3 probably by activating Smad2 and Smad3. In this study, we found that over-expressing ALK5 increased the expression of Smad3, but not that of Smad2. Smad2 and Smad3 genes were silenced by siRNA transfection to further explore the role of these endogenous genes in JEG-3 cell invasion. Silencing Smad3, not Smad2, inhibited the TGF-β1-induced cell invasion in JEG-3 cells. These results indicate that the TGF-β/Smad3 signaling pathway is involved in the effect of TGF-β1 on JEG-3 cell invasion.

The expression of two invasive-associated genes, namely, MMP-9 and MMP-2, increased after the TGF-β1 treatment. The stimulatory effect of TGF-β1 on these genes was enhanced by over-expressing TβRI but inhibited by SB431542. Inhibition of Smad3 and Smad2, especially Smad3, decreased the expression of MMP-9 and MMP-2 significantly. Furthermore, over-expressing Smad3 enhanced the expression of MMP-9 and MMP-2, whereas knockdown of Smad3 showed the opposite effect. Therefore, TGF-β/Smad3 signaling is involved in the up-regulation of MMP-9 and MMP-2 in JEG-3 after TGF-β1 treatment. MMPs are known to be the main mediators of extracellular matrix degradation [36–38]. MMP-9 and MMP-2 are abundantly expressed in invading EVT cells [39], and the expression of these two gelatinases is highly related to trophoblast cell invasiveness [40]. The invasive property of trophoblast cells could be attributed to their ability to degrade the extracellular matrix by secreting MMPs. Based on these results, we deduced that TGF-β1 promotes the invasion of JEG-3 by up-regulating the expression of MMP-9 and MMP-2.

The results indicate that the TGF-β/Smad3 pathway is involved in the stimulatory action of TGF-β1 on the invasion of JEG-3 cells. This study presents the first evidence of the key role of Smad3 in TGF-β1-induced JEG-3 cell invasion. Therefore, targeting Smad3 signaling might be a potential therapeutic approach for controlling the invasive function of JEG-3 cells. The present findings may provide new insights into the molecular mechanisms underlying the effect of TGF-β1 on regulating EVTs invasion and may contribute to the improvement of treatments for pregnancy diseases associated with abnormal trophoblast invasion.

## MATERIALS AND METHODS

### Cell culture and treatment

The human choriocarcinoma JEG-3 cells (Cell Resource Center, IBMS, CAMS/PUMC) were maintained in RPMI 1640 (Sigma) supplemented with 10% fetal bovine serum (FBS) and 1% Pen-Strep/100 U/ml penicillin, and 100 mg/ml streptomycin (Sigma) at 37°C in humidified 5% CO2 atmosphere. Cells were seeded at a concentration of 1 × 10^5^ /ml and the media were refreshed every 2 days. When cells were grown to approximately 60–70% confluence, the media were replaced with serum-free RPMI1640. After 24 h, the culture media were refreshed again and the cells were treated with various concentrations (0, 5, 10 or 20 ng⁄ml) of human recombinant TGF-β1 (PeproTech) for 48 h. In the case of inhibition assay, SB431542 (Sigma) at a concentration of 10μmol/L was supplemented to the culture 30 min before TGF-β1 treatment.

### Transfection experiments

JEG-3 cells were transfected with siRNA (chemically synthesized by Shanghai GenePharma, Shanghai, China) using RNAi-Mate (Shanghai GenePharma, Shanghai, China) following the manufacturer's protocol. The sequences of the siRNAs are available in Table 1. In the plasmid transfection experiments, JEG-3 cells were transfected with pCMV5 TBRI-HA (Addgene Plasmid 19162), CS2 Flag-Smad3 (EPSM) (Addgene Plasmid 14963), or the empty vector pCMV5 (Addgene) served as negative control using Lipofectamine^™^ 2000 (Invitrogen, Inc. Carlsbad,CA) according to the manufacturer's instructions. The time when transfection commenced was considered as time 0. After incubation in medium containing transfection reagent for 6h, the media were changed into normal growth medium. The cells were allowed to be recovered for 48 h, and then subjected to TGF-β1 or SB431542 treatment, mRNA quantification, protein analysis or invasion assay.

**Table 1:** Sequences of siRNAs targeting Smad2 and Smad3

SiRNA		Sequence (5′ to 3′)
Smad2-1	Sense	5′- CCAGGAAUAGCUAAAGAGAAGUCTT -3′
	Antisense	5′- AAGACUUCUCUUUAGCUAUUCCUGGUU -3′
Smad2-2	Sense	5′- GGCUGAGGUGGGAGGGUUACUUGGA -3′
	Antisense	5′- UCCAAGUAACCCUCCCACCUCAGCCUU -3′
Smad2-3	Sense	5′- GUACCAUUAAGAUGUGUGUUUCATG -3′
	Antisense	5′- CAUGAAACACACAUCUUAAUGGUACCA -3′
Smad3-1	Sense	5′- GUAUGUGCCUGGUGUGAAAUGAUCT -3′
	Antisense	5′- AGAUCAUUUCACACCAGGCACAUACUU -3′
Smad3-2	Sense	5′- CUGCCACCUUCAAUUGGUACUUUAT -3′
	Antisense	5′- AUAAAGUACCAAUUGAAGGUGGCAGUU -3′
Smad3-3	Sense	5′- CUACCAGAGAGUAGAGACACCAGTT -3′
	Antisense	5′- AACUGGUGUCUCUACUCUCUGGUAGUG -3′

### Transwell invasion assay

In the invasion assay, the BD Falcon cell culture inserts with polycarbonate filters (8μm pores) pre-coated with Matrigel were used. JEG-3 cells with different treatments were suspended in serum-free media at the density of 3 × 10^5^/ml and were plated into the upper chamber 200μl per well. The lower chamber was filled with 500 μl of media containing 10% FBS and 2 ng/ml VEGF (Peprotech). After incubation at 37°C, 5% CO2 for 24 h, media containing non-invaded cells were removed from the upper chamber and the cells remained in the upper surface of the filters were removed using cotton swabs. The lower surface of the filters with invaded cells attached were washed with PBS, fixed in 4% paraformaldehyde and stained with 0.1% crystal violet solution or VECTASHIELD Mounting Medium with DAPI (4′, 6-diamidino-2-phenylindole) (Vector Labs, Burlingame, CA). Cells were then identified and counted under an inverted light microscope in five random fields on high magnification. The average for the fields was used as the value for the well. Three independent experiments were performed with each condition being tested in triplicate.

### RNA isolation and reverse transcription-polymerase chain reaction (RT-PCR)

Total RNAs were isolated from JEG-3 cells using Trizol reagent (Invitrogen) and was reverse-transcribed into cDNA using the ReverTra Ace kit (Toyobo, Osaka, Japan) according to the manufacturer's instruction. Real-time quantitative PCR was carried out in a 20 μl reaction containing 0.6 μl of cDNA using the Eppendorf Mastercycler ep realplex real-time PCR system. After 15 min denaturation at 95°C, 40 cycles of amplification were carried out: 20 sec at 94°C, 10 sec at 58°C, and 15 sec at 72°C. Glyceraldehyde-3-phosphate dehydrogenase (GAPDH) was used as an internal control in each sample and the amount of cDNA was normalized against that of GAPDH. Negative controls in which cDNA sample was replaced with PCR grade water for each primer pair were included in each run. Specificity was confirmed by melting curve analysis and agarose gel electrophoresis. The sequences of gene-specific primers were listed in Table 2.

**Table 2:** Primers for real-time PCR

primers		Sequences	Size
MMP-2	F	5′-AGAAGGATGGCAAGTACGGCTTCT-3′	125
	R	5′-AGTGGTGCAGCTGTCATAGGATGT-3′	
MMP-9	F	5′-ATTTCTGCCAGGACCGCTTCTACT-3	87
	R	5′-TGTCATAGGTCACGTAGCCCACTT-3′	
Smad2	F	5′-TCTGCTCGAGAAGCCAGAATGTGT-3′	115
	R	5′-TCAGTCTGCATCAGGACACCCAAT-3′	
Smad3	F	5′-AGTGCTGGTGACTGGATAGCAGTT-3′	113
	R	5′- GCACAAGCTGCAAGGTGAAGATGT-3′	
Smad4	F	5′-TGTCCACAGGACAGAAGCCATTGA	105
	R	5′-TCACTAAGGCACCTGACCCAAACA	
Smad7	F	5′-GAAGCAGAAATCCAAGCACCACCA-3′	89
	R	5′- ACACTCACACTCACACACACTCCT-3′	
GAPDH	F	5′-AGCCTCAAGATCATCAGCAATGCC-3′	105
	R	5′-TGTGGTCATGAGTCCTTCCACGAT-3′	

### Western blot analysis

Nuclear proteins and cytoplasm proteins were extracted using Nuclear Extraction Kit (Panomics) following the manufacturer's protocol. Equal amount of protein lysates were separated by 10% SDS-PAGE, and transferred onto a polyvinylidene fluoride (PVDF) membrane. After incubation with the blocking solution (Tris-buffered saline containing 0.1% Tween-20 and 5% skim milk) at room temperature for 1h, the membrane was incubated with monoclonal rabbit anti-phospho-Smad2, antiphospho-Smad3, anti-Smad2, anti-Smad3, anti-Smad4, and anti-Smad7(1:1000 dilution, Epitomics), anti-MMP2, anti-MMP9 (1:200; Epitomics) at 4°C overnight. An antibody against β-actin, GAPDH, or α-tubulin (Earthox) was used as loading control for normalization of protein expression. Subsequently, the membrane was washed and then incubated with horseradish peroxide-conjugated antibody (1:5000, Boster, Wuhan, China) for 1h at room temperature. After extensive washing, the protein bands were detected using Gel Doc 2000^™^ gel documentation systems (Bio-Rad).

### Statistical analysis

Statistical analyses were performed using SPSS software (version 16.0). Data from at least three independent experiments performed in triplicate were expressed as mean ± SD and analyzed with a Student's *t-test* at a *P* < 0.05 level of significance.

## SUPPLEMENTARY MATERIALS FIGURES


