# Supplementary material for: Transforming growth factor β1 promotes invasion of human JEG-3 trophoblast cells via TGF-β/Smad3 signaling pathway
Source: Oncotarget. 2017 Apr 4;8(20):33560–70. doi: 10.18632/oncotarget.16826 (PMC5464890; doi:10.18632/oncotarget.16826)
Supplement: Supplementary file 1 [file oncotarget-08-33560-s001.pdf]

Transforming growth factor β1 promotes invasion of human JEG-3 trophoblast cells via TGF-β/Smad3 signaling pathway

Supplementary Materials

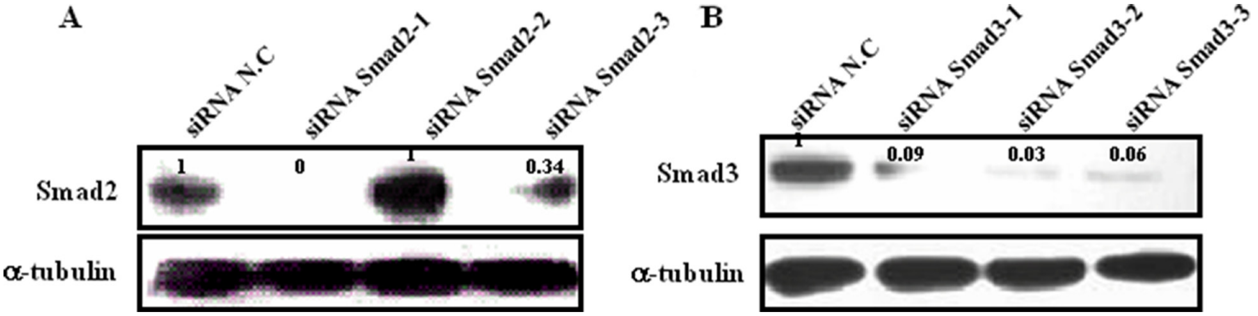

Supplementary Figure 1: Silence of Smad2 and Smad3 by siRNAs transfection. JEG-3 cells were transfected with siRNAs targeting Smad2 (siRNA Smad 2-1,-2,-3) or Smad3 (siRNA Smad3-1,-2,-3), respectively. Knockdown of Smad2 and Smad3 by siRNAs was confirmed by Western blot. (**A**) The most potent interference efficacy on blocking Smad2 expression appeared following Smad2-1 siRNA transfection. (**B**) The most potent interference efficacy on blocking Smad3 expression appeared following Smad3-2 siRNA transfection.
